# Case Report: Airway obstruction as a fatal complication of infantile hemangioma in newborns: the eye cannot see what the mind does not know

**DOI:** 10.3389/fped.2025.1631906

**Published:** 2025-09-30

**Authors:** Xiaoliang Liu, Li Ma, Fan Ma, JiaoJiao Wan, Huanrui Hu, Kaiyu Zhou, Fan Hu

**Affiliations:** ^1^ Key Laboratory of Birth Defects and Related Diseases of Women and Children of MOE, Department of Pediatrics, West China Second University Hospital, Sichuan University, Chengdu, Sichuan, China; ^2^ Department of Pediatrics, West China Second University Hospital-Tianfu·Sichuan Provincial Children's Hospital, Meishan, Sichuan, China; ^3^ Division of Vascular Surgery, Department of General Surgery, West China Hospital, Sichuan University, Chengdu, Sichuan, China

**Keywords:** infantile hemangioma, airway obstruction, propranolol, transcatheter interventional sclerotherapy, newborn

## Abstract

**Background:**

In newborns, infantile hemangiomas (IHs) in the airway can present with rare life-threatening complications associated with airway obstruction, even without cutaneous hemangioma.

**Case report:**

Our female newborn exhibited stridor 1 day after her birth. Furthermore, 7 days after birth, she was admitted to the local hospital to receive phototherapy for neonatal jaundice. However, her stridor was not noted as being related to IH by the clinician during her hospitalization. She was transferred to our hospital at 14 days old because her stridor and condition deteriorated with severe breathlessness and respiratory failure. Computed tomography and flexible bronchoscopy identified obstructive airway IHs. Although a combination therapy of propranolol and corticosteroids was prescribed for 2 weeks, she still needed invasive mechanical ventilation. After a discussion by the multidisciplinary team, endoscopic resection was carried out to resolve her airway obstruction at 30 days old, but it failed. Finally, transcatheter interventional sclerotherapy successfully resolved her clinical conditions at 60 days old. She was discharged and received follow-up with propranolol treatment. Her clinical symptoms of airway IHs were completely resolved 1 year later.

**Conclusions:**

Airway IHs can rarely present with life-threatening conditions associated with airway obstruction without cutaneous hemangioma in newborns. This case report highlights that propranolol is the cornerstone treatment for airway IHs. For the first time, our findings suggest that transcatheter interventional sclerotherapy can be a valid additional treatment option.

## Introduction

An infantile hemangioma (IH) is the most common pediatric soft-tissue tumor, with an incidence of 2%–10%. In newborns, its prevalence is approximately 5% (1). The tumor follows a lifecycle of rapid proliferation in infancy (the proliferative phase), followed by slow involution in childhood (the involution phase). IHs usually arise within 2–7 weeks of life in infancy. The early proliferative phase, characterized by fast growth, occurs during the ﬁrst 3–5 months. The late proliferative phase, characterized by slower growth, is usually completed by 9–12 months of age, but growth may continue beyond 36 months. The involution phase starts at approximately 12 months of age and lasts for 3–9 years, leaving residual changes in up to 69% of patients (2–4). A histological study of an IH in the proliferative phase revealed ongoing endothelial differentiation, with inchoate vessels expressing the endothelial markers VEGFR2 and CD31. During the proliferative phase, the majority of hemangioma vessels express GLUT1 as a hallmark, while significantly decreased expression of GLUT1 is found in GLUT1^+^ vessels during the involution phase (5). Therefore, GLUT1 expression is a diagnostic tool for an IH.

Although IHs yield no serious risk, 10%–15% of IHs lead to complications and are referred to as high-risk IHs. According to the clinical practice guideline for the management of infantile hemangiomas by the American Academy of Pediatrics, high-risk IHs can lead to disfigurement, life-threatening complications, functional impairment, ulceration, and underlying abnormalities (6). For patients with high-risk IHs, the life-threatening complications include obstructive airway hemangiomas, liver hemangiomas, cardiac failure, and hypothyroidism. Clinically, close follow-up is crucial in early life, as the majority of cases of IH do not require therapy. Moreover, patients with high-risk IHs require timely and prompt management. The guidelines recommend oral propranolol as the first-line treatment for problematic IHs that require systemic therapy. Surgery and/or laser therapy are commonly recommended for residual skin changes during the involution phase in IHs (6).

Airway IHs are more likely to involve the larynx or upper airway of the subglottic region (7). They can lead to respiratory distress and biphasic stridor and should be differentiated from inﬂammatory croup, reﬂux, subglottic obstruction, intubation granuloma, cysts, congenital cricoid malformation, and the presence of a foreign body (8). Moreover, considering their rapid proliferation in infancy, airway IHs can present with life-threatening complications associated with airway obstruction, with an incidence of 1.4% (9). Airway IHs infiltrate the intraluminal or extraluminal bronchus, causing compression and hyperinflation of the affected side and subsequently resulting in acute respiratory failure (7, 10–12). Therefore, it is of great importance for clinicians to recognize that airway IHs can result in airway obstruction. These patients may require urgent airway intervention via a tracheostomy (13). Oral propranolol, alone or combined with systemic corticosteroids, is the first-line therapy for airway IHs (6, 7, 14).

Herein, for the first time, we report a rare, life-threatening case of IH with airway obstruction that was not resolved using an initial combination therapy of propranolol and corticosteroids but was finally resolved via transcatheter interventional sclerotherapy. Our findings suggest that clinicians need to recognize and promptly manage airway IHs in newborns. Furthermore, our findings highlight that propranolol is the cornerstone treatment for airway IHs, and transcatheter interventional sclerotherapy is a valid, additional treatment option.

## Case presentation

A female newborn was delivered at the local hospital with a birth weight of 3,680 g at a gestational age of 39^+1^ weeks. After delivery, her Apgar score was normal with 10-10-10, and there was no history of intrauterine distress. There were no indications of a premature rupture of membranes. The patient’s parents noted that she exhibited mild stridor without accompanying hoarseness or dyspnea 1 day after birth. Furthermore, 7 days after birth, she was admitted to the local hospital for hyperbilirubinemia. On arrival, the physical examination found jaundice involving the entire body and normal body temperature, heart rate, respiratory rate, and percutaneous arterial oxygen saturation (SpO_2_). The stridor was noted. Other physical findings were negative. Laboratory tests identified hyperbilirubinemia with elevated levels of total serum bilirubin (TBil; 360.7 µmol/L, normal range < 342 µmol/L) and hemoglobin (Hb; 186 g/L, normal range: 140–170 g/L), and normal results in the other auxiliary examinations. The patient was discharged home with a normal TBil level 5 days later after receiving phototherapy. Regrettably, her stridor was not noted as being related to IHs by the clinician during her hospitalization and was considered a symptom of laryngomalacia. However, the patient’s parents noted that her stridor was more severe at home.

**Figure 1:**
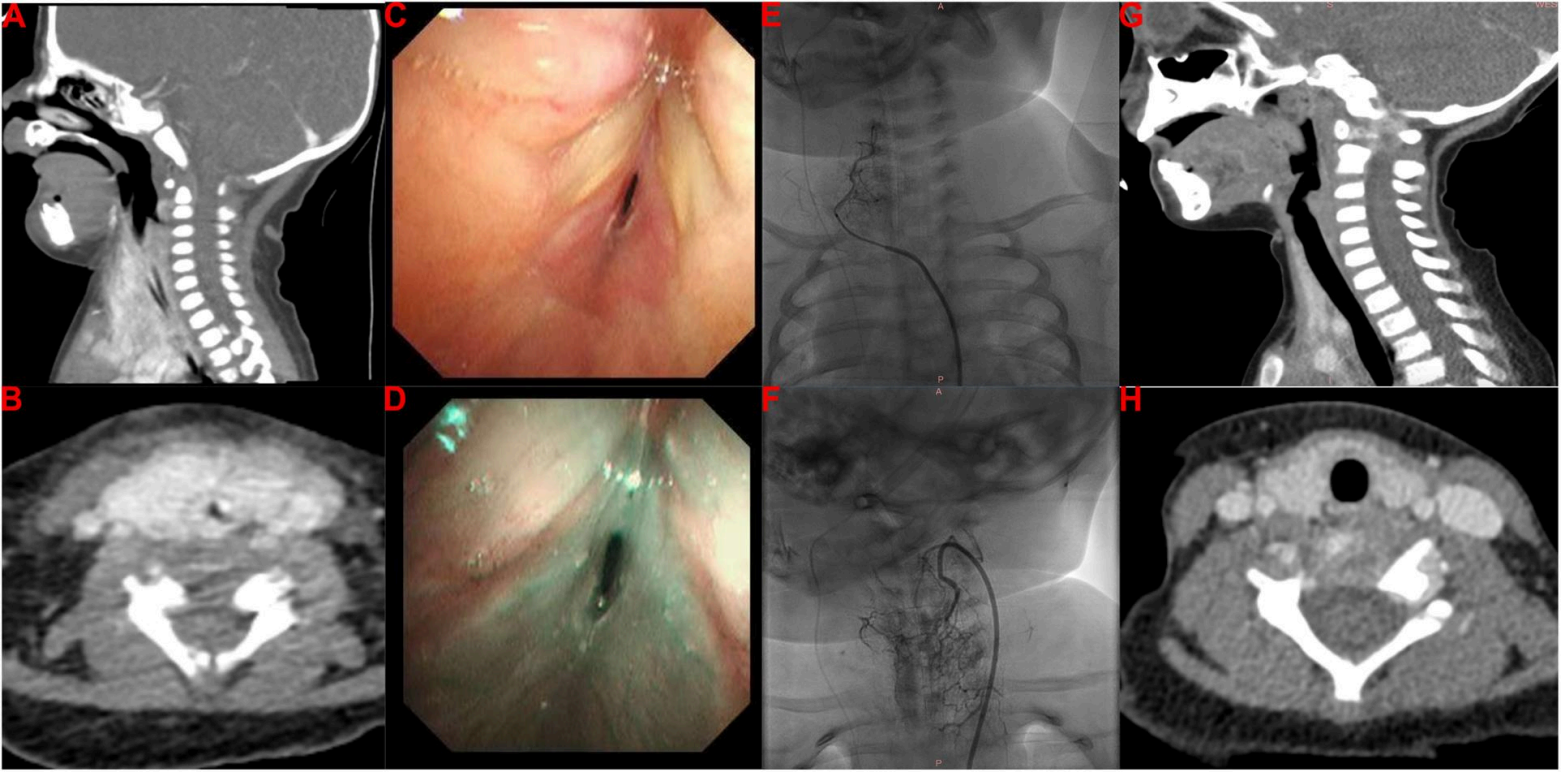
Imaging findings of our patient with infantile hemangioma with airway obstruction. (**A,B**) Contrast-enhanced CT imaging reveals a thickening of circumferential tissue with contrast enhancement around the subglottic area. (**C,D**) Flexible bronchoscopy identifies IHs in the airway and an airway obstruction. (**E,F**) Transcatheter interventional sclerotherapy is performed. (**G,H**) Repeated contrast-enhanced CT reveals that the circumferential tissue thickening with contrast enhancement around the subglottic area was resolved with the treatment.

She was then transferred to our hospital to investigate the cause of the stridor in the larynx at 14 days old. On arrival, the physical examination found stridor with abnormal airway sounds, nasal flaring, head bobbing, and increased accessory muscle use. The patient’s respiratory rate was 70–80/min, her SaO_2_ was 88%–92%, and her heart rate was 160/min. The findings indicated that her clinical condition had deteriorated with severe breathlessness and progression to respiratory failure. Due to the early onset and persistence of the stridor and her progressive breathlessness, the congenital causes of stridor due to a structural abnormality were initially proposed, including laryngomalacia, tracheomalacia, vocal cord paresis/paralysis, vascular ring, bronchogenic cyst, laryngeal malformations, IHs, and subglottic stenosis. Contrast-enhanced computed tomography (CT) revealed circumferential tissue thickening with contrast enhancement around the subglottic area (Figures 1A,B), indicating the possibility of IH. Furthermore, flexible bronchoscopy identified airway IHs and an airway obstruction (Figures 1C,D). Due to the patient’s clinical manifestations and the imaging findings, the clinician proposed a diagnosis of airway IHs, which accounted for her deteriorating clinical condition. The guideline for the management of IH by the American Academy of Pediatrics strongly recommends clinicians use oral propranolol as the first-line therapy for IHs requiring systemic treatment (grade A, strong recommendation) (6). A surgical intervention may be recommended as a treatment option in selected IHs (grade C, moderate recommendation) (6). Notably, with the advent of β-blocker therapy, surgical interventions are used less frequently. The previous gold standard therapy for life-threatening IHs was systemic or intralesional corticosteroids (7, 15, 16). Therefore, corticosteroid therapy is also recommended for IHs in specific settings, such as in patients with IHs who present with contraindicated, poorly tolerated, or ineffective β-blocker therapy. Complete informed consent regarding the treatment plan was obtained from the patient’s legal guardian, and they chose a combination therapy of oral propranolol and corticosteroids. The combination therapy of oral propranolol (gradually increased to 2 mg/kg twice a day) and corticosteroids (2 mg/kg twice a day) was prescribed for 2 weeks after the multidisciplinary team's discussion. Regrettably, she still required invasive mechanical ventilation after this 2-week therapy. After the multidisciplinary team's discussion, endoscopic resection was carried out by a pediatric otolaryngologist, aiming to resolve her airway obstruction at 30 days old; however, this surgical intervention failed. We then explained the patient's current condition and prognosis to the patient's guardian in detail. Finally, her guardian agreed to transcatheter interventional sclerotherapy (Figures 1E,F). The feeding arteries of the lesions were embolized using penile vascular anomalies (PVAs) (300 µm) and iodinated oil to reduce the blood flow rate. Bleomycin (0.5 mg/kg) was injected as the sclerotherapeutic agent. Encouragingly, this additional treatment resolved the patient’s clinical symptoms, and the invasive mechanical ventilation was withdrawn 7 days later. At 60 days old, she was discharged home and received follow-up at our outpatient clinic, undergoing oral propranolol therapy. One year later, a neck CT identified that the circumferential tissue thickening with contrast enhancement around the subglottic area had been resolved (Figures 1G,H), and she did not have any clinical symptoms of airway obstruction.

## Discussion

IHs are benign self-limiting tumors in infancy; however, airway IHs can result in airway obstruction as a life-threatening complication (7, 9). It is widely recognized that oral propranolol is the first-line therapy for airway IHs that require systemic treatment (6, 7, 14). In this case report, we report a rare, life-threatening case of IH with airway obstruction that was not resolved by the initial combination therapy of propranolol and corticosteroids. The patient underwent respiratory support with invasive mechanical ventilation for more than 1 month. Encouragingly, her clinical condition finally resolved after receiving transcatheter interventional sclerotherapy. Our findings suggest that it is crucial for clinicians to promptly recognize and manage airway IHs in infants who are at high risk of airway obstruction. Moreover, this case report highlights that transcatheter interventional sclerotherapy is a valid additional treatment option alongside propranolol, which is the cornerstone treatment for IHs with airway involvement.

**Figure 2:**
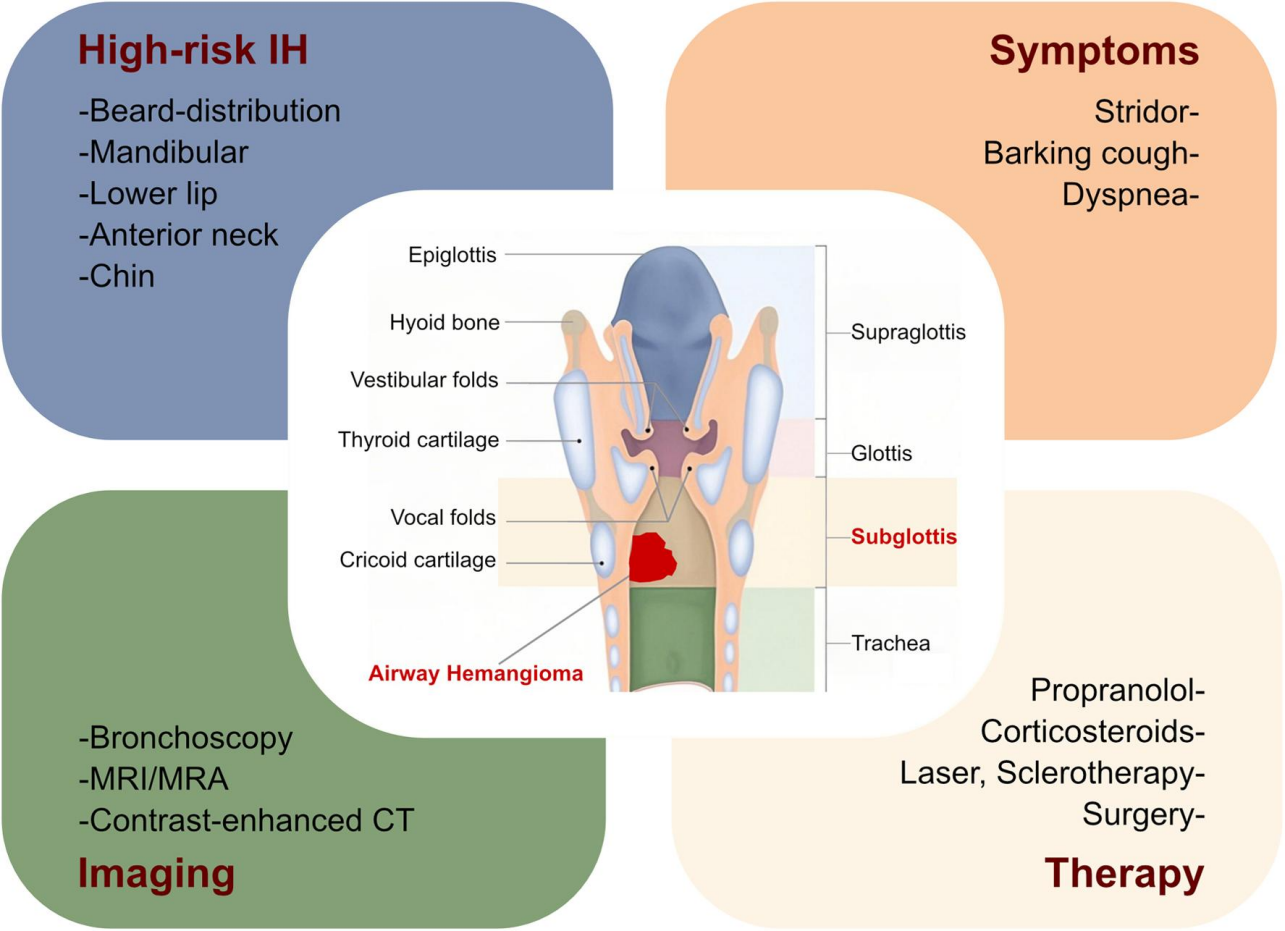
Summary of airway infantile hemangioma. Herein, we summarize the risk of obstructive airway hemangioma in patients with IHs, along with their clinical symptoms, imaging examinations, and therapies. MRI, magnetic resonance imaging; MRA, magnetic resonance angiography; CT, computed tomography; IH, infantile hemangioma.

Airway obstruction is a symptom in patients with airway IHs and subsequently contributes to respiratory insufficiency and life-threatening complications. Therefore, it is crucial for clinicians to recognize the skin patterns that indicate a high risk for airway IHs. Clinicians have explored the possible association between hemangiomas in the upper airway or subglottic regions and facial hemangiomas. Orlow et al. evaluated the association between cutaneous cervicofacial IHs in a “beard” distribution and symptomatic hemangiomas in the upper airway or subglottic areas and revealed that IHs in a beard distribution indicated the possibility of upper airway or subglottic involvement (17, 18). Piram et al. also investigated the association between cutaneous IHs and subglottic IHs and found that the IHs in a median pattern, IHs in the neck area, and the telangiectatic IH subtype were more likely to be accompanied by subglottic IH involvement (19). In addition, Uthurriague et al. reported 38 patients with airway IHs from multiple centers, the majority of whom were accompanied by a cutaneous or mucosal hemangioma. They found that patients with a large segmental mandibular hemangioma were at high risk for airway IHs and segmental lower lip and neck involvement (20). These findings suggest that patients with segmental hemangiomas in a cervicofacial, mandibular, or “beard” distribution, comprising the preauricular skin, mandible, lower lip, chin, or anterior neck, were at high risk of developing airway IHs (17–21). In this case, our patient did not have a cutaneous or mucosal hemangioma. The patient’s stridor, the initial clinical symptom of airway IHs, occurred after birth and subsequently progressed to breathlessness and respiratory failure. Regrettably, the clinician did not recognize the possibility of airway IHs as the cause of the stridor, which may partly be attributed to the absence of a cutaneous hemangioma. In clinical settings, the incidence of airway IHs with no skin involvement is low (20), making it a diagnostic challenge, as the clinical symptom of stridor could be due to other causes. Notably, a subglottic hemangioma is the most common neoplasm found in infants’ airways (16), with stridor the most common clinical manifestation before 6 months of age. Other subglottic diseases should also be considered, including inﬂammatory croup, reﬂux, subglottic obstruction, intubation granuloma, cysts, and congenital cricoid malformation (8). The clinical presentation of airway IHs is characterized by speciﬁc timing and is significantly indicative, manifesting as progressive airway obstruction after birth within the proliferation stage. After the first year of disease onset, patients with this disorder enter the involution stage and the symptoms gradually resolve. Therefore, previous findings and this case strongly suggest that clinicians should suspect airway IHs in infants presenting with the rapid development of respiratory symptoms, such as stridor. These patients may further benefit from early recognition and prompt management (Figure 2).

Although airway IH is a benign disease, it can be life-threatening. It was reported that the mortality rate ranged from 30% to 70% among patients without a timely diagnosis and prompt management (22, 23). Clinicians should suspect the diagnosis of airway IHs depending on the patient’s disease history, clinical manifestations, and physical examination, and the diagnosis can be confirmed by an endoscopic examination and/or imaging characteristics (16). The color of airway IHs in the endoscopic examination varies from red to blue, depending on the thickness of the overlying mucosa and degree of vascularity. A biopsy is rarely required. The most common location of subglottic hemangioma is on the left side, followed by circumferential, bilateral, unilateral, and with or without posterior extension (16). It has been reported that asymmetrical narrowing of the subglottic airway on frontal neck radiographs is pathognomonic for hemangioma (24, 25). However, only 50% of subglottic hemangiomas present with asymmetrical narrowing, with the remaining 50% presenting with symmetrical narrowing (26). It is recommended that magnetic resonance imaging (MRI) and/or magnetic resonance angiography (MRA) be performed if there is suspicion of cervical or intrathoracic extension of the hemangioma (6, 23, 27). Dynamic, contrast-enhanced CT is not the modality of choice for imaging IHs because of ionizing radiation; however, a CT examination is a valuable, non-invasive method and can be rapidly performed (28). Koplewitz et al. reported that a CT examination can be used to confirm a diagnosis of IH in equivocal cases instead of the invasive endoscopic examination (28). In this study, an MRA examination was primarily suggested but was refused by the patient’s guardian because of the long examination time, and an enhanced CT was finally performed to image the lesions and confirm the diagnosis (28). Notably, it is critical to determine the optimal imaging and sedation protocols through a multidisciplinary discussion (6, 29).

The intervention approach for IHs depends on the risk stratification. Patients with high-risk IHs, including those with (1) life-threatening complications, (2) functional impairment or ulceration, (3) structural anomalies, or (4) permanent disfigurement, need early and prompt management, as a wait-and-see approach might lead to adverse outcomes (6). The US Food and Drug Administration (FDA) has approved oral propranolol hydrochloride for patients with proliferating IHs that require systemic therapy. The mechanisms of propranolol treatment mainly include vasoconstriction, angiogenesis inhibition, induction of apoptosis, inhibition of nitric oxide production, and regulation of the renin-angiotensin system (30). Oral propranolol demonstrated superior efficacy when compared with other interventions (topical timolol, intralesional triamcinolone, and oral steroid), with a mean expected clearance of 95% (27). The recommended propranolol dose is 2–3 mg/kg/day, and a lower dose should be considered in the presence of comorbidities (e.g., PHACE syndrome) or adverse effects (e.g., sleep disturbances, bronchial irritation, and/or clinically symptomatic bradycardia and hypotension). Notably, to reduce the risk of hypoglycemia, propranolol should be administered with or after feeding (31, 32). In addition, the administration of corticosteroids for IHs should be proposed when patients with IHs have contraindications or an inadequate response to propranolol (7, 15, 16). If patients have focal, bulky IHs during the proliferation phase or IHs in specific critical anatomical locations, such as the lip, sclerotherapy should be considered (33, 34). Patients with thin and/or superficial IHs can be treated with topical timolol maleate (6, 35, 36).

Early identification of airway IHs facilitates early management, which leads to an improved prognosis. It is strongly recommended that clinicians use oral propranolol as the first-line therapy for IHs requiring systemic treatment (6, 37). Notably, the corticosteroid therapy is also recommended for IHs in specific settings, such as in patients with IHs who present with contraindicated, poorly tolerated, or ineffective β-blocker therapy. After the advent of β-blocker therapy, laser approaches have been used less, while surgical therapy may be recommended as a treatment option in selected patients with IHs, including those with ulceration and/or airway obstruction. Previous studies have also outlined open excision and laryngotracheoplastic approaches in patients with subglottic hemangiomas, which cause subglottic narrowing of more than 70% and bilateral or circumferential lesions (16). Only a few case reports have described microlaryngoscopic ablation with a CO_2_ laser as an optional treatment for airway IHs (38, 39). In line with the guidelines, our patient received a combination therapy of oral propranolol and corticosteroids for 2 weeks. However, she still required invasive mechanical ventilation. After the multidisciplinary team's discussion, an endoscopic resection with a CO_2_ laser was performed, but it failed. Finally, transcatheter interventional sclerotherapy was performed and successfully resolved her clinical symptoms at 60 days old. She was discharged home 2 weeks later and received follow-up at our outpatient clinic, undergoing oral propranolol therapy. The clinical symptoms associated with this patient’s IHs were completely resolved 1 year later, as identified by neck CT.

## Conclusion

In clinical settings, it is crucial for clinicians to recognize and manage IHs in the airways of infants. This case highlights that propranolol, alone or combined with corticosteroids, is the cornerstone treatment for airway IHs. Our findings suggest, for the first time, that transcatheter interventional sclerotherapy is a valid additional treatment option.

## Data Availability

The original contributions presented in the study are included in the article/Supplementary Material, further inquiries can be directed to the corresponding author.

## References

[1] Holm A Mulliken JB Bischoff J. Infantile hemangioma: the common and enigmatic vascular tumor. J Clin Invest. (2024) 134(8):e172836. 10.1172/jci172836 38618963 PMC11014660

[2] Rodríguez Bandera AI Sebaratnam DF Wargon O Wong LF. Infantile hemangioma. Part 1: epidemiology, pathogenesis, clinical presentation and assessment. J Am Acad Dermatol. (2021) 85(6):1379–92. 10.1016/j.jaad.2021.08.019 34419524

[3] Tollefson MM Frieden IJ. Early growth of infantile hemangiomas: what parents’ photographs tell us. Pediatrics. (2012) 130(2):e314–20. 10.1542/peds.2011-3683 22826568

[4] Luu M Frieden IJ. Haemangioma: clinical course, complications and management. Br J Dermatol. (2013) 169(1):20–30. 10.1111/bjd.12436 23701395

[5] Huang L Nakayama H Klagsbrun M Mulliken JB Bischoff J. Glucose transporter 1-positive endothelial cells in infantile hemangioma exhibit features of facultative stem cells. Stem Cells. (2015) 33(1):133–45. 10.1002/stem.1841 25187207 PMC4270824

[6] Krowchuk DP Frieden IJ Mancini AJ Darrow DH Blei F Greene AK Clinical practice guideline for the management of infantile hemangiomas. Pediatrics. (2019) 143(1):e20183475. 10.1542/peds.2018-3475 30584062

[7] Corbeddu M Meucci D Diociaiuti A Giancristoforo S Rotunno R Gonfiantini MV Management of upper airway infantile hemangiomas: experience of one Italian multidisciplinary center. Front Pediatr. (2021) 9:717232. 10.3389/fped.2021.717232 34950613 PMC8688849

[8] Ida JB Thompson DM. Pediatric stridor. Otolaryngol Clin North Am. (2014) 47(5):795–819. 10.1016/j.otc.2014.06.005 25213283

[9] Haggstrom AN Drolet BA Baselga E Chamlin SL Garzon MC Horii KA Prospective study of infantile hemangiomas: clinical characteristics predicting complications and treatment. Pediatrics. (2006) 118(3):882–7. 10.1542/peds.2006-0413 16950977

[10] Sacco O Moscatelli A Nozza P Rossi GA. Respiratory distress in a 3-month-old infant with a mass obstructing the right main-stem bronchus: an unusual localization of infantile hemangioma. J Pediatr. (2017) 182:397–e1. 10.1016/j.jpeds.2016.11.063 28017308

[11] Sierpina DI Chaudhary HM Walner DL Aljadeff G Dubrow IW. An infantile bronchial hemangioma unresponsive to propranolol therapy: case report and literature review. Arch Otolaryngol Head Neck Surg. (2011) 137(5):517–21. 10.1001/archoto.2011.67 21576565

[12] MacDougall MS Afzal SY Freedman MS Han P. Infant in extremis: respiratory failure secondary to lower airway infantile hemangioma. BMC Pediatr. (2022) 22(1):744. 10.1186/s12887-022-03821-1 36581920 PMC9801545

[13] Diociaiuti A Baselga E Boon LM Dompmartin A Dvorakova V El Hachem M The VASCERN-VASCA working group diagnostic and management pathways for severe and/or rare infantile hemangiomas. Eur J Med Genet. (2022) 65(6):104517. 10.1016/j.ejmg.2022.104517 35487416

[14] Truong MT Chang KW Berk DR Heerema-McKenney A Bruckner AL. Propranolol for the treatment of a life-threatening subglottic and mediastinal infantile hemangioma. J Pediatr. (2010) 156(2):335–8. 10.1016/j.jpeds.2009.10.010 20105647

[15] Greene AK Couto RA. Oral prednisolone for infantile hemangioma: efficacy and safety using a standardized treatment protocol. Plast Reconstr Surg. (2011) 128(3):743–52. 10.1097/PRS.0b013e3182221398 21572374

[16] Rahbar R Nicollas R Roger G Triglia JM Garabedian EN McGill TJ The biology and management of subglottic hemangioma: past, present, future. Laryngoscope. (2004) 114(11):1880–91. 10.1097/01.mlg.0000147915.58862.27 15510009

[17] Orlow SJ Isakoff MS Blei F. Increased risk of symptomatic hemangiomas of the airway in association with cutaneous hemangiomas in a “beard” distribution. J Pediatr. (1997) 131(4):643–6. 10.1016/s0022-3476(97)70079-9 9386676

[18] Ezekowitz RA. The relationship between facial and airway hemangiomas: does seeing red bode ill? J Pediatr. (1997) 131(4):514–5. 9386650

[19] Piram M Hadj-Rabia S Boccara O Couloigner V Hamel-Teillac D Bodemer C. Beard infantile hemangioma and subglottic involvement: are median pattern and telangiectatic aspect the clue? J Eur Acad Dermatol Venereol. (2016) 30(12):2056–9. 10.1111/jdv.13812 27406622

[20] Uthurriague C Boccara O Catteau B Fayoux P Léauté-Labrèze C Chiaverini C Skin patterns associated with upper airway infantile haemangiomas: a retrospective multicentre study. Acta Derm Venereol. (2016) 96(7):963–6. 10.2340/00015555-2357 26832659

[21] Mackey WS. Infantile hemangioma with a focus on airway hemangioma. ORL Head Neck Nurs. (2016) 34(2):18–23. 27305734

[22] Brodsky L Yoshpe N Ruben RJ. Clinical-pathological correlates of congenital subglottic hemangiomas. Ann Otol Rhinol Laryngol. (1983) 105:4–18. 10.1177/00034894830920s401 6410970

[23] Mulliken JB Fishman SJ Burrows PE. Vascular anomalies. Curr Probl Surg. (2000) 37(8):517–84. 10.1016/s0011-3840(00)80013-1 10955029

[24] Sutton TJ Nogrady MB. Radiologic diagnosis of subglottic hemangioma in infants. Pediatr Radiol. (1973) 1(4):211–6. 10.1007/bf00972854 4779071

[25] Holinger LD Toriumi DM Anandappa EC. Subglottic cysts and asymmetrical subglottic narrowing on neck radiograph. Pediatr Radiol. (1988) 18(4):306–8. 10.1007/bf02388997 3387150

[26] Cooper M Slovis TL Madgy DN Levitsky D. Congenital subglottic hemangioma: frequency of symmetric subglottic narrowing on frontal radiographs of the neck. AJR Am J Roentgenol. (1992) 159(6):1269–71. 10.2214/ajr.159.6.1442399 1442399

[27] Chinnadurai S Snyder K Sathe N Fonnesbeck C Morad A Likis FE Diagnosis and management of infantile hemangioma. Agency Healthc Res Qual. (2016). PMID: 26889533

[28] Koplewitz BZ Springer C Slasky BS Avital A Uwyyed K Piccard E CT of hemangiomas of the upper airways in children. AJR Am J Roentgenol. (2005) 184(2):663–70. 10.2214/ajr.184.2.01840663 15671395

[29] Borghi L. Practice advisory on anesthetic care for magnetic resonance imaging: an updated report by the American Society of Anesthesiologists Task Force on anesthetic care for magnetic resonance imaging. Anesthesiology. (2015) 122(3):495–520. 10.1097/aln.0000000000000458 25383571

[30] Ji Y Chen S Xu C Li L Xiang B. The use of propranolol in the treatment of infantile haemangiomas: an update on potential mechanisms of action. Br J Dermatol. (2015) 172(1):24–32. 10.1111/bjd.13388 25196392

[31] Holland KE Frieden IJ Frommelt PC Mancini AJ Wyatt D Drolet BA. Hypoglycemia in children taking propranolol for the treatment of infantile hemangioma. Arch Dermatol. (2010) 146(7):775–8. 10.1001/archdermatol.2010.158 20644039

[32] Breur JM de Graaf M Breugem CC Pasmans SG. Hypoglycemia as a result of propranolol during treatment of infantile hemangioma: a case report. Pediatr Dermatol. (2011) 28(2):169–71. 10.1111/j.1525-1470.2010.01224.x 20738795

[33] Chen MT Yeong EK Horng SY. Intralesional corticosteroid therapy in proliferating head and neck hemangiomas: a review of 155 cases. J Pediatr Surg. (2000) 35(3):420–3. 10.1016/s0022-3468(00)90205-7 10726680

[34] Prasetyono TO Djoenaedi I. Efficacy of intralesional steroid injection in head and neck hemangioma: a systematic review. Ann Plast Surg. (2011) 66(1):98–106. 10.1097/SAP.0b013e3181d49f52 21042190

[35] Püttgen K Lucky A Adams D Pope E McCuaig C Powell J Topical timolol maleate treatment of infantile hemangiomas. Pediatrics. (2016) 138(3):e20160355. 10.1542/peds.2016-0355 27527799

[36] Chakkittakandiyil A Phillips R Frieden IJ Siegfried E Lara-Corrales I Lam J Timolol maleate 0.5% or 0.1% gel-forming solution for infantile hemangiomas: a retrospective, multicenter, cohort study. Pediatr Dermatol. (2012) 29(1):28–31. 10.1111/j.1525-1470.2011.01664.x 22150436

[37] Blei F. Oral prednisolone for infantile hemangioma: efficacy and safety using a standardized treatment protocol. Plast Reconstr Surg. (2012) 129(5):840e–1e. 10.1097/PRS.0b013e31824a61a7 22544114

[38] Mesolella M Allosso S Mansueto G Fuggi M Motta G. Strategies and controversies in the treatment with carbon dioxide laser of laryngeal hemangioma: a case series and review of the literature. Ear Nose Throat J. (2022) 101(5):326–31. 10.1177/0145561320952191 32921178

[39] Almothahbi A Bukhari M Almohizea M Alsubaie N Alharbi TF Alhazzani HM Recent updates in laryngeal hemangioma management: a scoping review. Eur Arch Otorhinolaryngol. (2024) 281(5):2211–22. 10.1007/s00405-023-08378-y 38158419

